# Infestation by *Ornithodoros rietcorreai* on captive *Boa constrictor* with reports of human parasitism and environmental colonization

**DOI:** 10.1590/S1984-29612026010

**Published:** 2026-06-15

**Authors:** Josenilton Rodrigues Santos, Ila Ferreira Farias, Glauber Menezes Barboza de Oliveira, Kayo Eduardo de Andrade Lima, Rafael dos Santos Dantas, Marcelo Bahia Labruna, Mauricio Claudio Horta

**Affiliations:** 1 Universidade Federal do Vale do São Francisco – UNIVASF, Laboratório de Doenças Parasitárias, Petrolina, PE, Brasil; 2 Universidade Federal do Vale do São Francisco – UNIVASF, Programa de Pós-graduação em Ciências Veterinárias no Semiárido, Petrolina, PE, Brasil; 3 Parque Zoobotânico da Caatinga – 72º BICaat, Petrolina, PE, Brasil; 4 Universidade de São Paulo – USP, Faculdade de Medicina Veterinária e Zootecnia, Departamento de Medicina Veterinária Preventiva e Saúde Animal, Laboratório de Doenças Parasitárias, São Paulo, SP, Brasil

**Keywords:** Argasidae, tick, jiboia, anthropophilia, Caatinga, semiarid, Argasidae, carrapato, jibóia, antropofilia, Caatinga, semiárido

## Abstract

Reptiles of the order Squamata are commonly parasitized by ticks in the family Ixodidae. However, they can also host argasid ticks of the genus *Ornithodoros*. In this study, we reported *Ornithodoros rietcorreai* parasitizing a *Boa constrictor* and a human in Caatinga Zoobotanical Park, within the 72^nd^ Caatinga Infantry Battalion of the Brazilian Army in Petrolina, Pernambuco (PE). Thirty argasid tick specimens, including two larvae, 17 nymphs, four females, and seven males, were removed from the body surface of the snake. Substantial environmental infestations were observed within the snake enclosure, with ticks in the soil, beneath rocks, and on wood. In total, 126 tick specimens comprising 70 larvae, 13 nymphs, 17 females, and 26 males were collected from the environment. During the sampling process, a team member that was parasitized by larvae developed edema, erythema, and pruritus, at the bite sites after 24 h. To our knowledge, this is the first study to report parasitism by *O. rietcorreai* in a captive *Boa constrictor* in Brazil, along with a case of human parasitism within a snake enclosure.

## Resumo

Os répteis da ordem Squamata são habitualmente parasitados por carrapatos da família Ixodidae. Contudo, também podem ser parasitados por argasídeos do gênero *Ornithodoros.* Neste estudo, relatamos *Ornithodoros rietcorreai* parasitando *Boa constrictor* e humano no Parque Zoobotânico da Caatinga, localizado dentro do 72º Batalhão de Infantaria da Caatinga do Exército Brasileiro em Petrolina, Pernambuco (PE). Um total de 30 espécimes de Argasídeos, incluindo duas larvas, 17 ninfas, quatro fêmeas e sete machos foram retirados da superfície corporal da serpente. Além disso, uma infestação ambiental significativa foi observada dentro do recinto da serpente, com carrapatos encontrados no solo, sob pedras e na madeira. Um total de 126 espécimes compreendendo 70 larvas, 13 ninfas, 17 fêmeas e 26 machos foram coletados no ambiente. Durante o processo de coleta de amostras, um membro da equipe foi parasitado por larvas, desenvolvendo edema, eritema e prurido nos locais das picadas após 24 horas. Este estudo é o primeiro a relatar parasitismo por *O. rietcorreai* em uma jiboia em cativeiro no Brasil, juntamente com um caso de parasitismo humano dentro do recinto da serpente.

*Boa constrictor constrictor* Linnaeus, 1758, is a reptile from the order Squamata and family Boidae, known for its nocturnal and semi-arboreal habits. This species occurs throughout Central and South America, including all Brazilian biomes such as the Caatinga (Zug et al., 2001; Grego et al., 2014). Owing to their non-venomous nature and conservation efforts, the Boidae family is highly valued for captive breeding, making them popular pets (Bérnils & Costa, 2012; Grego et al., 2014). Maintaining this subspecies in captivity requires careful feeding, appropriate management, and suitable facility conditions. However, common prophylactic measures are often inadequate to ensure snake health. Therefore, they are vulnerable to opportunistic infections and infestations (Barbosa et al., 2006; Pereira et al., 2012).

Boa constrictors are commonly parasitized by ixodid ticks of the genus *Amblyomma*, including *Amblyomma dissimile* Koch, 1844*, Amblyomma fuscum* Neumann, 1907, and *Amblyomma rotundatum* Koch, 1844 (Carrascal et al., 2009; Dantas-Torres et al., 2008, 2010; Pereira et al., 2012; Bastos et al., 2016; Fiorini et al., 2014). Captive boas live in confined areas that provide ectoparasites the opportunity to establish themselves in the environment (Krebber Mogollón et al., 2017). The scarcity of information on argasid parasites infesting ectothermic animals in Northeast Brazil makes it relevant to improve studies by describing new host-parasite associations (Dantas-Torres et al., 2008). This is particularly relevant considering the single report of parasitism by *Ornithodoros* sp. on a captive *B. constrictor constrictor* in Rio Grande do Norte (Pereira et al., 2012).

**Figure 1:**
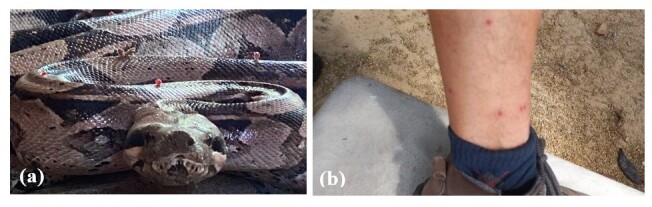
Parasitism by *Ornithodoros rietcorreai* on *Boa constrictor constrictor* (a) and on a human (b).

In this study, we have reported intense parasitism by argasid ticks on a captive *Boa constrictor* from the Caatinga Zoobotanical Park (9º 22’ 58” S 40º 28’ 56” W), located within the 72^nd^ Caatinga Infantry Battalion of the Brazilian Army in Petrolina, PE. We have also included a report on human parasitism and environmental colonization. During routine management in March 2024, the boa was found parasitized by argasid ticks along its dorsal body surface (Figure 1a). Many ticks were found in the soil, under rocks, and on wood in the enclosure. The ticks were collected using anatomical tweezers, placed in tubes containing 70% ethanol, and transported to the laboratory. During sampling, a park team member noticed numerous larvae climbing on their clothing, followed by discomfort due to bites on their lower limbs, indicating human parasitism (Figure 1b). Within 24 h, edema, erythema, and pruritus appeared at the tick bite sites.

The larvae were mounted on semi-permanent slides in Hoyer’s medium and examined under an optical microscope. Nymphs and adults were morphologically examined under a stereomicroscope. Ticks of all stages were morphologically identified to species according to original descriptions (Labruna et al., 2016). All the ticks were identified as *Ornithodoros rietcorreai* Labruna, Nava & Venzal, 2016. The characters considered for identifying *O. rietcorreai* larvae were: 14 pairs of dorsal setae, pear-shaped dorsal plate, hypostome with pointed apex and dental formula 3/3 anteriorly, 2/2 posteriorly, and anal valves with long, pointed, leaf-shaped ends. The characters of nymphs and adults were confirmed by the presence of large, clustered, irregularly shaped, slightly raised nipples with short hairs, generally one or two hairs per nipple. Distinct discs, covered by flattened nipples in slightly depressed areas over a median area, with marginal grooves present, differentiating females from males through the semilunar-shaped genital opening and the genital opening with a semicircular flap, at the level of coxa I, respectively.

There were 30 specimens in parasitism, comprising two larvae, 17 nymphs, four females, and seven males, and 126 free-living specimens, including 70 larvae, 13 nymphs, 17 females, and 26 males. The identified ticks were deposited in the tick collection “Coleção Nacional de Carrapatos Danilo Gonçalves Saraiva” (CNC) of the Faculty of Veterinary Medicine of the University of São Paulo, and in the tick collection of the Laboratory of Parasitic Diseases of UNIVASF.

The affected boa was treated with a single topical application of fipronil (Shooter spray®) at a dose of 7.5 mg/kg, equivalent to 3 mL/kg according to the manufacturer. The enclosure was subjected to fire sweeping to control the ectoparasites. Infestation was likely initiated by the presence of infested rodents and/or other synanthropic animals in the forests near the enclosures.

The family Argasidae comprises five genera, namely, *Argas*, *Antricola*, *Otobius*, *Nothoaspis*, and *Ornithodoros* (Nava et al., 2017). *Ornithodoros rietcorreai* is one of 24 species of argasid ticks found in Brazil (Labruna et al., 2026). First described in the states of Paraíba and Piauí in 2016, *O. rietcorreai* occurs in the Caatinga biome and is associated with the habitats of wild rodents such as the cavy (*Galea spixii*) and the rock cavy (*Kerodon rupestris*). To a lesser extent, it is also associated with bats living in rocky cavities in northeastern Brazil (Labruna et al., 2016; Jorge et al., 2022). Parasitism by ticks of the genus *Ornithodoros* on captive *B. constrictor* was also recorded at the Onélio Porto Zoobotanical Park at UFERSA in Mossoró, RN. However, the ticks could not be identified to species level (Pereira et al., 2012). Infestation by *O. rietcorreai* at their larval stage has also been observed on the snake *Leptodeira annulata* (Alcantara et al., 2018). In this study, parasitism by larvae, nymphs, and adults of *O. rietcorreai* on *B. constrictor* was confirmed. This suggests that, despite the strong association of ticks with wild rodents, snakes can also be important hosts for maintaining parasite colonies. The parasitism of *O. rietcorreai* on boa constrictors is not so unexpected, since this species belongs to the subgenus *Alectorobius*. In fact, *Ornithodoros puertoricensis* Fox, 1947, which also belongs to this subgenus, was reported to parasitize a monitor lizard and two species of python, and at a level of infestation that demonstrated that reptiles were suitable hosts (Bermúdez et al., 2015). This information is even more relevant when considering the most recent taxonomic classification proposed by Mans et al. (2021), that elevate this subgenus to the level of genus.

Nine *Ornithodoros* species have been reported parasitizing humans in Brazil, highlighting the issue of parasitism and the potential for pathogen transmission. This emphasizes the importance of these agents to public health (Nogueira et al., 2022). *Ornithodoros rietcorreai* is found in urban environments, causing toxicosis in humans, and has already proven positive in molecular analyses for microorganisms with unknown pathogenicity (Oliveira et al., 2018; Muñoz-Leal et al., 2019, 2021).

Our findings confirm parasitism by *O. rietcorreai* on a captive *B. constrictor*, along with reports of human parasitism within snake enclosure. The presence of all the active stages of the tick life cycle in the boa and the enclosure suggests that the boa was able to maintain a population of *O. rietcorreai* under captive conditions. Understanding the interspecific relationships between captive and wild animals, and the potential sharing of parasites, is essential. These interspecific relationships can harm animals by transmitting pathogenic agents to other animals and humans. Therefore, a deeper understanding is beneficial in the context of health, wellbeing, and species conservation.

## References

[B001] Alcantara EP, Ferreira-Silva C, Ávila RW, Pacheco RC, Martins TF, Muñoz-Leal S (2018). Ticks (Acari: Argasidae and Ixodidae) infesting amphibians and reptiles in Northeastern Brazil. Syst Appl Acarol.

[B002] Barbosa AR, Silva H, Albuquerque HN, Ribeiro IAM (2006). Contribuição ao estudo parasitológico de jibóias, *Boa constrictor* Linnaeus, 1758, em cativeiro. Rev Biol Ciênc Terra.

[B003] Bastos TSA, Madrid DMC, Faria AM, Freitas TMS, Linhares GFC (2016). Carrapatos em animais silvestres do bioma cerrado triados pelo Cetas, IBAMA-Goiás. Cienc Anim Bras.

[B004] Bermúdez S, Miranda RJ, Cleghorn J, Venzal JM (2015). *Ornithodoros* (*Alectorobius*) *puertoricensis* (Ixodidae: Argasidae) parasitizing exotic reptiles in Panama. FAVE Secc Cienc Vet.

[B005] Bérnils RS, Costa HC (2012). Brazilian reptiles: list of species. Version 2012.1.

[B006] Carrascal JV, Oviedo TS, Monsalve SB, Torres AM (2009). *Amblyomma dissimile* (Acari: Ixodidae) parásito de *Boa constrictor* en Colombia. Rev MVZ Cordoba.

[B007] Dantas-Torres F, Ferreira DRA, Melo LM, Lima PCP, Siqueira DB, Rameh-de-Albuquerque LC (2010). Ticks on captive and free-living wild animals in northeastern Brazil. Exp Appl Acarol.

[B008] Dantas-Torres F, Oliveira-Filho EF, Soares FÂM, Souza BOF, Valença RBP, Sá FB (2008). Ticks infesting amphibians and reptiles in Pernambuco, Northeastern Brazil. Rev Bras Parasitol Vet.

[B009] Fiorini LC, Craveiro AB, Mendes MC, Chiesorin L, Silveira RD (2014). Morphological and molecular identification of ticks infesting *Boa constrictor* (Squamata, Boidae) in Manaus (Central Brazilian Amazon). Rev Bras Parasitol Vet.

[B010] Grego KF, Albuquerque LR, Kolesnikovas CKM, Cubas ZS, Silva JCR, Catão-Dias JL (2014). Tratado de animais selvagens: medicina veterinária.

[B011] Jorge FR, de Oliveira LMB, Magalhães MML, Weck B, de Oliveira GMB, Serpa MCA (2022). New records of soft ticks (Acari: Argasidae) in the Caatinga biome of Brazil, with a phylogenetic analysis of argasids using the nuclear *Histone 3* (H3) gene. Exp Appl Acarol.

[B012] Krebber Mogollón HR, Mestra Pineda A, Herrera Benavides YM, Causil Vargas L (2017). *Amblyomma dissimile* en *Boa constrictor* en cautiverio del Centro de Atención y Valoración de Fauna Silvestre de Montería (Córdoba, Colombia). Rev Med Vet (Bogota).

[B013] Labruna MB, Barros-Battesti DM, Martins TF, Muñoz-Leal SA (2026). Argasidae in Catálogo Taxonômico da Fauna do Brasil.

[B014] Labruna MB, Nava S, Marcili A, Barbieri ARM, Nunes PH, Horta MC (2016). A new argasid tick species (Acari: Argasidae) associated with the rock cavy, *Kerodon rupestris* Wied-Neuwied (Rodentia: Caviidae), in a semiarid region of Brazil. Parasit Vectors.

[B015] Mans BJ, Kelava S, Pienaar R, Featherston J, de Castro MH, Quetglas J (2021). Nuclear (18S-28S rRNA) and mitochondrial genome markers of *Carios* (*Carios*) *vespertilionis* (Argasidae) support *Carios* Latreille, 1796 as a lineage embedded in the Ornithodorinae: re-classification of the *Carios* sensu Klompen and Oliver (1993) clade into its respective subgenera. Ticks Tick Borne Dis.

[B016] Muñoz-Leal S, Faccini-Martinez AA, Teixeira BM, Martins MM, Serpa MCA, Oliveira GMB (2021). Relapsing fever group borreliae in human-biting soft ticks, Brazil. Emerg Infect Dis.

[B017] Muñoz-Leal S, Macedo C, Gonçalves TC, Barreira JD, Labruna MB, de Lemos ERS (2019). Detected microorganisms and new geographic records of *Ornithodoros rietcorreai* (Acari: Argasidae) from northern Brazil. Ticks Tick Borne Dis.

[B018] Nava S, Venzal JM, Acuña DG, Martins TF, Guglielmone AA (2017). Ticks of the Southern Cone of America: diagnosis, distribution, and hosts with taxonomy, ecology and sanitary importance..

[B019] Nogueira BCF, Campos AK, Muñoz-Leal S, Pinter A, Martins TF (2022). Soft and hard ticks (Parasitiformes: Ixodida) on humans: a review of Brazilian biomes and the impact of environmental change. Acta Trop.

[B020] Oliveira SV, Bitencourth K, Borsoi ABP, de Freitas FSS, Coelho GCB, Amorim M (2018). Human parasitism and toxicosis by *Ornithodoros rietcorreai* (Acari: Argasidae) in an urban area of Northeastern Brazil. Ticks Tick Borne Dis.

[B021] Pereira JS, Dias CEV, Filgueira TMB, Freitas CIA, Ahid SMM (2012). Infestação por carrapatos em *Boa constrictor* (Linnaeus,1758) de cativeiro, em Mossoró, Rio Grande do Norte. Rev Bras Zoociênc.

[B022] Zug GR, Vitt LJ, Caldwell JP, Zug GR, Vitt LJ, Caldwell JP (2001). Herpetology: an introductory biology of amphibians and reptiles..

